# Associations between White Matter Hyperintensities and β Amyloid on Integrity of Projection, Association, and Limbic Fiber Tracts Measured with Diffusion Tensor MRI

**DOI:** 10.1371/journal.pone.0065175

**Published:** 2013-06-06

**Authors:** Linda L. Chao, Charles DeCarli, Stephen Kriger, Diana Truran, Yu Zhang, Joel Laxamana, Sylvia Villeneuve, William J. Jagust, Nerses Sanossian, Wendy J. Mack, Helena C. Chui, Michael W. Weiner

**Affiliations:** 1 Department of Radiology & Biomedical Imaging, University of California San Francisco, San Francisco, California, United States of America; 2 Center for Imaging of Neurodegenerative Diseases, Veterans Affairs Medical Center, San Francisco, San Francisco, California, United States of America; 3 Department of Neurology, University of California Davis, Davis, California, United States of America; 4 Helen Wills Neuroscience Institute, University of California, Berkeley, California, United States of America; 5 School of Public Health, University of California, Berkeley, California, United States of America; 6 Department of Neurology, University of Southern California, Los Angeles, California, United States of America; 7 Department of Preventive Medicine, University of Southern California, Los Angeles, California, United States of America; University of Manchester, United Kingdom

## Abstract

The goal of this study was to assess the relationship between Aβ deposition and white matter pathology (i.e., white matter hyperintensities, WMH) on microstructural integrity of the white matter. Fifty-seven participants (mean age: 78±7 years) from an ongoing multi-site research program who spanned the spectrum of normal to mild cognitive impairment (Clinical dementia rating 0–0.5) and low to high risk factors for arteriosclerosis and WMH pathology (defined as WMH volume >0.5% total intracranial volume) were assessed with positron emission tomography (PET) with Pittsburg compound B (PiB) and magnetic resonance and diffusion tensor imaging (DTI). Multivariate analysis of covariance were used to investigate the relationship between Aβ deposition and WMH pathology on fractional anisotropy (FA) from 9 tracts of interest (i.e., corona radiata, internal capsule, cingulum, parahippocampal white matter, corpus callosum, superior longitudinal, superior and inferior front-occipital fasciculi, and fornix). WMH pathology was associated with reduced FA in projection (i.e., internal capsule and corona radiate) and association (i.e., superior longitudinal, superior and inferior fronto-occipital fasciculi) fiber tracts. Aβ deposition (i.e., PiB positivity) was associated with reduced FA in the fornix and splenium of the corpus callosum. There were interactions between PiB and WMH pathology in the internal capsule and parahippocampal white matter, where Aβ deposition reduced FA more among subjects with WMH pathology than those without. However, accounting for apoE ε4 genotype rendered these interactions insignificant. Although this finding suggests that apoE4 may increase amyloid deposition, both in the parenchyma (resulting in PiB positivity) and in blood vessels (resulting in amyloid angiopathy and WMH pathology), and that these two factors together may be associated with compromised white matter microstructural integrity in multiple brain regions, additional studies with a longitudinal design will be necessary to resolve this issue.

## Introduction

Focal and diffuse lesions in the white matter, seen as hyperintensities on T2-weighted magnetic resonance images (MRI) (i.e., white matter hyperintensities; WMH) are the most ubiquitous age-related alteration seen in the brain [1]. From an etiological perspective, heterogeneity exists with respect to the presence of WMH. On one hand, they are related to cardiovascular risk factors, such as hypertension and atherosclerosis [2]–[4]. On the other hand, they are abundantly present in patients with an underlying amyloid pathology such as cerebral amyloid angiopathy (CAA) [5]. Another common pathological change in the aging brain is cerebral β-amyloid (Aβ) deposition. Aβ is the major component of amyloid plaques and has been hypothesized to initiate a pathogenic cascade that eventually leads to Alzheimer’s disease (AD) [6]. Aβ deposition and vascular brain injury frequently coexist [7], [8]. We recently showed that aggregate coronary disease risk has a moderately strong association with cerebral amyloid deposition [9], consistent with the theory that vascular risk factors (e.g., low high density lipoportein) contribute to AD pathology [10]. The goal of this study was to assess the relationship between Aβ deposition and WMH pathology, defined as greater than 0.5% of total intracranial volume (ICV) [11], on microstructural integrity of the white matter.

Diffusion tensor imaging (DTI) measures the organization of fibers within specific white matter tracts [12]. An indirect measure of the coordinated directionality and coherence of fibers within a white matter fiber bundle [13], fractional anisotropy (FA) values typically decrease in the presence of pathology such as stroke [14] and WMH [15]. Here we used DTI to determine the regional pattern of FA disruption associated with WMH pathology and Aβ deposition. Based on previous DTI studies in patients with AD and mild cognitive impairment (MCI), a prodromal phase of AD, we hypothesized that Aβ deposition would be associated with decreased FA in the limbic circuit [16]–[20] (e.g., cingulum, parahippocampal white matter, and fornix) and posterior regions of the corpus callosum [21], [22]. Because the anterior internal capsule and supratentorial white matter are common locations for WMH in the human brain [23], we hypothesized that WMH pathology would be associated with lower FA in projection (e.g., internal capsule and corona radiata) and association (e.g., superior longitudinal, superior and inferior fronto-occipital fasciculi) fiber tracts and in the corpus callosum, the major white matter structure involved in intrahemispheric cortico-cortical communication.

Recent epidemiologic studies have noted that risk factors for atherosclerosis (e.g., diabetes mellitus, hypertension, and hyperlipidemia) are also associated with increased risk of incident AD [24], [25]. Moreover, it has been suggested that vascular brain injury may interact with concomitant AD pathology [26]. For this reason, we hypothesized that there would be an interaction between Aβ deposition and WMH pathology on FA. In particular, we looked for a Aβ deposition by WMH pathology interaction in the corpus callosum because Lee et al [27]. previously reported differential region-specific associations between AD degenerative and vascular processes on the structural integrity of the corpus callosum.

## Methods

### Subjects

Subjects were older adults from the ongoing multi-site Aging Brain research program designed to recruit individuals with substantial risk factors for arteriosclerosis and vascular brain injury [9]. Persons with history of multiple vascular risk factors, coronary or carotid disease, myocardial infarction, or ischemic stroke were targeted for inclusion. Exclusion criteria included evidence of alcohol or substance abuse, head trauma with loss of consciousness lasting longer than 15 minutes, factors contraindicating MRI, and severe medical illness, neurologic or psychiatric disorders unrelated to AD or vascular dementia that could significantly affect brain structure (e.g., schizophrenia and other psychotic disorders, liver disease, multiple sclerosis, amyotrophic lateral sclerosis).

This study focused on a subset of 57 individuals who had PiB PET and DTI data. The mean time between acquisition of the PiB and DTI imaging data was 3.9±4.9 months (range: 0–19 months). The subjects received clinical diagnostic evaluations at the Alzheimer’s Disease Centers at the University of California at Davis and San Francisco. For purposes of analyses, the participants were classified by their Clinical Dementia Rating (CDR [28]) scores. Twenty-nine subjects (51%) had CDRs of 0 while 28 (49%) had CDRs of 0.5. Table 1 provides more details on participant characteristics.

**Table 1:** Sample characteristics by PIB and WMH pathology^a^ status.

	PIB−	PIB+	WMH−	WMH+	PiB effect	WMH effect
	(N = 30)	(N = 27)	(N = 30)	(N = 27)	p-value	p-value
Age (yrs)	77.3 (7.3)	79.0 (6.6)	77.0 (7.0)	79.1 (6.9)	0.76^d^	0.22^d^
Sex (M/F)	20/10	20/7	23/7	17/10	0.54^e^	0.26^e^
Education (yrs)	14.3 (3.0)	14.7 (3.1)	14.6 (3.3)	14.4 (2.7)	0.67^d^	0.84^d^
MMSE	28.5 (2.0)	28.1 (2.0)	28.3 (2.0)	28.2 (2.0)	0.49^d^	0.84^d^
GDS	1.4 (1.8)	2.4 (2.7)	2.0 (2.6)	1.8 (1.9)	0.12^d^	0.76^d^
CDR 0/0.5	19/11	10/17	16/14	13/14	0.047^e^	0.70^e^
apoE ε 4 (−/+)^b^	21/5	13/9	19/7	15/7	0.10^e^	0.71^e^
Global PiB index	1.00 (0.00)	1.26 (0.45)	1.17 (0.38)	1.10 (0.27)	0.001^d^	0.30^d^
PiB −/+			16/14	14/13		0.91^e^
Brain infarct on MRI	9 (30%)	13 (48%)	10 (33%)	12 (44%)	0.16^e^	0.39^e^
WMH^c^ volume as % ICV	0.50 (0.64)	0.81 (0.89)	0.20 (0.12)	1.29 (0.84)	0.23^d^	<0.001^d^
WMH pathology (−/+)	16/14	14/13			0.91^e^	

a Defined as WMH volume ≥0.5% of total intracranial volume (ICV).

b unavailable for 9 participants.

c derived from FLAIR segmentation, available for 24 PiB−, 26 PiB+, 28 WMH**−**, 22 WMH+.

d *p*-value from MANOVA, df = 1,55.

e *p*-value from Pearson Chi-Square test.

### Ethics Statement

The study was approved by the University of California (Davis, San Francisco, and Berkeley) and Lawrence Berkeley National Laboratory institutional review boards (IRB) for human research. Written informed consent was obtained from all participants or their legally authorized representatives following IRB-approved protocols. If there was an indication of cognitive impairment (e.g., in cases where the study participant had a CDR of 0.5 and/or a diagnosis of MCI), the study personnel obtaining consent would first assess whether or not the potential participant understood key elements of the protocol, including that participation is voluntary, that it is a research study, the procedures, the risks and benefits. If the potential participant lacked the capacity to provide informed consent, then informed consent was obtained from the participant’s legally authorized representative in accordance with California law and the policy of the IRB of all participating institutions. The majority of participants with CDRs of 0.5 had the capacity to provide informed consent.

### MRI Acquisition

MRI images were collected on Siemens 3 T MRI systems. Eight participants were scanned using a 3 T Siemens Magnetom Trio Syngo System with an 8-channel head coil at the University of California, Davis (UCD) research center. Acquired images included a T1-weighted volumetric MP-RAGE (TR = 2500, TE = 2.98, TI = 1100, 1×1×1 mm^3^ isotropic resolution), a Fluid-Attenuated Inversion Recovery (FLAIR) scan (TR = 5000, TE = 430, TI = 1700 ms, with 1×1×2 mm^3^ resolution), a T2-weighted turbospin echo sequence for intracrinal volume (ICV) determination (TR/TE: 3400/402 ms, 1×1×1 mm^3^ isotropic resolution), and DTI scans obtained with a dual spin-echo EPI sequence, augmented by diffusion weighting gradients (b = 800 s/mm2) along six collinear directions and the acquisition of a reference image (b = 0). Other parameters of DTI were TR/TE = 10,000/101 ms, 60 slices 2×2×2 mm^3^ resolution. Twofold parallel imaging acceleration was used for DTI to reduce geometrical distortions [29], and five scans were averaged to boost the signal.

Forty-three participants were scanned at UCD using a 3 T Siemens Magnetom TrioTim system with an 8-channel head coil. Six participants were scanned at the University of California, San Francisco (UCSF) Neuroscience Imaging Center using a 3 T Siemens Magnetom TrioTim system with a 12-channel head coil. Acquired images for the participants scanned on the 3 T Siemens Magnetom TrioTim system at UCD and UCSF included a T1-weighted volumetric MP-RAGE (TR = 2500, TE = 2.98, TI = 1100, 1×1×1 mm^3^ isotropic resolution), a FLAIR scan (TR = 8800, TE = 495, TI = 2360 ms, with 1×1×2 mm^3^ resolution), a T2-weighted turbospin echo sequence (TR/TE: 3400/402 ms, 1×1×1 mm^3^ isotropic resolution), and DTI scans obtained with a dual spin-echo EPI sequence supplemented with GRAPPA to reduce susceptibility distortions (TR/TE = 9000/101 ms, 60 slices 2×2×2 mm^3^ resolution; 5 scans averaged to boost the signal).

### Infarcts

Infarcts, identified by a vascular neurologist (NS) blind to any other participant data using the T1 and FLAIR MRIs, were identified on 22 of the 57 participants (39%). Infarcts were categorized according to affected vascular territory, structures involved, size (small 3–10 mm, large: >10 mm), severity (cystic, not cystic), and number. Table 2 presents a summary of the description of the participants’ infarcts.

**Table 2:** Description of infarcts in 22 participants.

Number of Infarcts	N
1 infarct	16
2 infarcts	5
4 infarcts	1
**Infarct size**	
Small (3–10 mm)	17
Large (>10 mm)	13
**Infarct severity**	
Cystic	22
Not cystic	8
**Vascular territory affected**	
Middle cerebral artery	15
Anterior cerebral artery	2
Posterior cerebral artery	3
Basilar artery	6
Posterior inferior cerebellar artery	4
**Hemisphere affected**	
Right	18
Left	12
**Infarct location**	
Frontal gray matter	6
Frontal white matter	7
Parietal gray matter	4
Parietal white matter	4
Temporal white matter	1
Subcortical gray matter^1^	13
Corpus callosum or internal capsule	4
Other^2^	11

1 basal ganglia and/or thalamus.

2 midbrain, pons, medulla and/or cerebellum.

### Apolipoprotein E Genotyping

Blood was drawn with the subject’s consent for apolipoprotein E (apoE) genotyping, available for 48 of the 57 participants. Subjects with 3/4 or 4/4 combined alleles were classified as apoE ε4 positive, and those with 3/3 alleles as apoE ε4 negative. Because the 2/4 combined allele is associated with a lower risk of AD [30], the one subject with 2/4 apoE genotype was not included in the apoE ε4 positive group.

### WMH Pathology

WMH were segmented from skull-stripped, intensity inhomogeneity corrected FLAIR images [31], [32]. The WMH lesion segmentations were visually inspected to ensure that the segmentations included at least 80% of observed WMH. This labor intensive process was only performed on participants with WMH lesions that appeared on 3 consecutive slices in all 3 orientations (50 of the 57 participants). Participants who did not meet this criterion were classified as not having WMH pathology. WMH pathology was operationalized as WMH volume greater than 0.5% of TIV (28 of the 57 participants).

### DTI Processing

Preprocessing of the DTI data involved skull-stripping, motion and eddy-current correction with FSL package, geometric distortion correction [33]. After the creation of FA images for each participant, an automated region of interest (ROI) extraction was performed. First, all FA images were resliced to the SPM8 white matter (WM) template using a rigid-body transformation (dimensions: 121×145×121 voxels, resolution: 1.5 mm^3^). Next, the ‘JHU ICBM-DTI-81’ atlas package [34], which includes a probabilistic FA atlas and anatomical labels of 50 deep WM ROIs from the DTI data of 81 subjects, was imported into SPM8, co-registered, and resliced to the SPM8 WM template. Next, the resliced FA images were normalized to an intensity-averaged FA template generated from 23 Aging Brain research participants (mean age 75±6; 8 female) who had no infarcts and whose WMH burden volume was less than 0.5% of intracranial volume (ICV), considered significant WMH pathology [11], using a diffeomorphic registration algorithm (DARTEL) [35]. Finally, the individual FA images and the ‘JHU ICBM-DTI-81’ FA atlas were warped to the common space of the averaged FA template generated from 23 VBI- participants, whose DTI data, other than use in creation of the intensity-averaged FA template, were not part of the current analyses. To avoid inclusion of surrounding gray matter or cerebral spinal fluid, DTI values were obtained only in voxels where FA was greater than 0.20.

**Figure 1:**
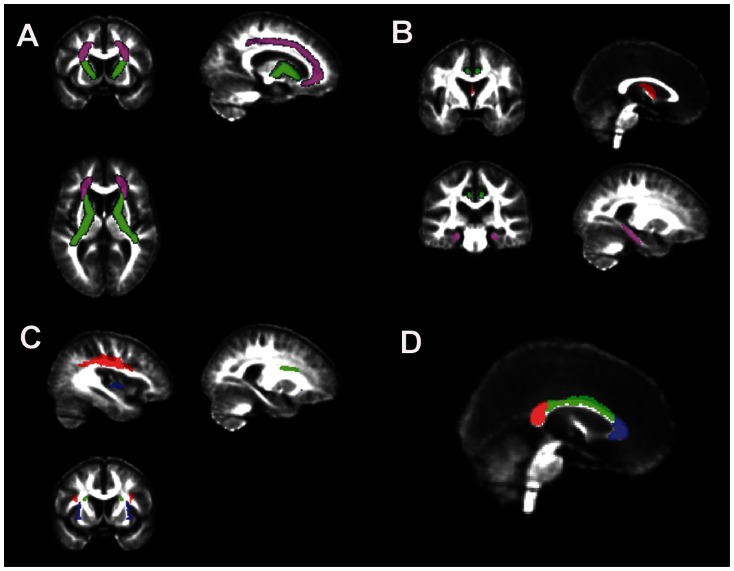
Examples of the (A) projection fiber ROIs: corona radiata in magenta; internal capsule in green, (B) limbic fiber ROIs: cingulate gyrus in green, parahippocampal white matter in magenta; fornix in red, (C) association fiber ROIs: superior longitudinal fasciculus in red; superior fronto-occipital fasciculus in green; inferior fronto-occipital fasciculus in purple, and (D) corpus callosum ROIs: genu in purple; body in green; splenium in red. ROIs are overlaid an intensity-averaged FA template generated from 23 Aging Brain research participants with no infarct and WMH <0.5% intracranial volume.

Based on the literature and a priori hypotheses, FA were extracted from 9 tracts of interest that included the corona radiata and internal capsule (projection fibers), cingulum gyrus and parahippocampal white matter, and fornix (limbic fibers), superior longitudinal fasciculus, inferior and superior fronto-occipital fasciculi (association fibers), and corpus callosum. Figure 1 shows examples of the a priori tracts of interest overlaid on the intensity-averaged FA template.

### PET: Acquisition

PiB-PET imaging was conducted at Lawrence Berkeley National Laboratory using a Siemens ECAT EXACT HR PET scanner in 3D acquisition mode. The PiB radiotracer was synthesized using a previously published protocol [36] and 10–15 mCi of PiB was injected as a bolus into an antecubital vein. Dynamic acquisition frames (34 or 35 frames total) were obtained over 90 minutes as following: 415 seconds, 830 seconds, 960 seconds, 2180 seconds, 8300 or 10300 seconds, and 3600 or 2600 seconds. For additional details see [37].

### PET: Processing and Analysis

PiB-PET data was preprocessed using the Statistical Parametric Mapping software package (SPM8; http://www.fil.ion.ucl.ac.uk/spm). Region of Interest (ROI) labeling, including the cerebellum gray matter masked, was implemented using the FreeSurfer v5.1 software package (http://surfer.nmr.mgh.harvard.edu/). Distribution volume ratio (DVR) images were created using Logan graphical analysis with PiB frames corresponding to 35–90 minutes post-injection and a gray matter cerebellar mask as the reference region [38], [39]. The grey matter cerebellum was chosen as the reference region because it is known to be relatively unaffected by fibrillar amyloid in AD and is thus valuable to estimate non-specific PiB binding [39], [40].

ROIs were defined in each subject’s native space using the Desikan-Killiany atlas and the semi-automated FreeSurfer processing stream [41]. A global measure of PiB uptake (Global PiB Index) was derived by averaging the mean DVR value from frontal (cortical regions anterior to the precentral gyrus), temporal (middle and superior temporal regions), parietal (supramarginal gyrus, inferior/superior parietal lobules and precuneus) and anterior and posterior cingulate ROIs. ROIs that overlapped with large cortical infarcts or cerebellar cortical infarcts were manually edited so that DVR values excluded these regions.

Subjects were classified as having high or low PiB uptake using a previously described cutoff approach [37]. That is, subjects were classified PiB positive if their global PiB uptake was 2 SD above (i.e., >1.08) of the mean PiB index value of 11 young subjects (age range = 20–30) unlikely to have amyloid deposition.

### Statistical Analyses

All analyses were conducted using IBM SPSS Statistics software (version 20.0). Associations among dichotomous variables were assessed using Pearson Chi-Square test. We used multivariate analysis of covariance (MANCOVA) to evaluate the effects of WMH pathology and PiB status on FA from the 9 a priori tracts of interest, controlling for age, CDR, and presence of infarct. Because we had no a priori hypotheses about laterality, FA from homologous parcels was averaged across hemispheres to reduce the number of comparisons. To further protect against type I error, we combined several of the ‘JHU ICBM-DTI-81’ white matter parcels (i.e., anterior, superior, and posterior regions of the corona radiata, anterior, posterior, and retrolenticular sections of the internal capsule, and 4 subregions of the cingulate gyrus). We examined the 3 subregions of the corpus callosum (i.e., genu, body, and splenium) separately because we wanted to test the patho-anatomic model for regional disruption of callosal FA proposed by Lee et al [27]. In secondary analyses, we re-evaluated the effects of WMH pathology and PiB status on FA from the 9 a priori tracts of interest in the subset of subjects who had no infarct(s) and controlled for the effects of apoE ε4 in the subset of subjects who had apoE genotype information.

## Results

### Participant Characteristics

Table 1 summarizes the demographic and clinical data of participants by PiB and WMH pathology status. There were no significant differences in age, sex, education, Mini-Mental State Examination (MMSE), Geriatric Depression Scale (GDS) scores, WMH volume, apoE ε4 status, or frequency of brain infarct among PiB negative (N = 30) and positive (N = 27) participants. However, more PiB positive subjects had CDR score of 0.5 (χ^2^ = 3.93, df = 1, p<0.05) than PiB negative. There were no significant age, sex, education, MMSE, GDS, CDR, apoE, global PiB index, or frequency of brain infarct among participants with (N = 27) or without (N = 30) WMH pathology. As expected, WMH volume was larger among participants with than without WMH pathology (F_1,48_ = 46.09, p = 0.001). Twenty-two participants (3 WMH**−**PiB−; 6 WMH+PiB−; 7 WMH**−** PiB+; 6 WMH+PiB+) had radiologic evidence infarct(s). A summary of the description of the participants’ infarcts is presented in Table 2.

Table 3 summarizes the effects of PiB positivity and WMH pathology on FA values from the 9 tracts of interest by group. After controlling for age, CDR, and presence of infarct(s), PiB positivity was associated with lower FA in the fornix (F_1,50_ = 6.69, p = 0.01) and the splenium of the corpus callosum (F_1,50_ = 4.20, p<0.05). WMH pathology was associated with lower FA in the corona radiata (F_1,50_ = 23.34, p<0.001), internal capsule (F_1,50_ = 23.53, p<0.001), superior longitudinal fasciculus (F_1,50_ = 12.72, p = 0.001), superior fronto-occipital fasciculus (F_1,50_ = 11.43, p = 0.001), inferior fronto-occipital fasciculus (F_1,50_ = 11.47, p = 0.001), and body of the corpus callosum (F_1,50_ = 7.9954, p = 0.01). There was a positive interaction between PiB and WMH pathology in the internal capsule (F_1,50_ = 6.39, p = 0.01) and parahippocampal white matter (F_1,50_ = 5.16, p = 0.03), where PiB-related reductions in FA was greater among participants with WMH pathology.

**Table 3:** Least Squares Means FA values from 9 ROIs by PiB and WMH pathology status^a^.

	PiB−WMH−	PiB−WMH+	PiB+WMH+	PiB+WMH+		p-value	
	(n = 16)	(n = 14)	(n = 14)	(n = 13)	PiB	WMH	PiB X WMH
**Projection Fibers**							
Corona radiata	0.40 (0.03)	0.38 (0.03)	0.41 (0.03)	0.36 (0.03)	0.38	<0.001	0.08
Internal capsule	0.51 (0.03)	0.50 (0.03)	0.53 (0.03)	0.48 (0.03)	0.65	<0.001	0.01
**Limbic Fibers**							
Cingulum	0.33 (0.03)	0.34 (0.03)	0.33 (0.03)	0.33 (0.03)	0.61	0.29	0.73
Parahippocampal WM	0.34 (0.03)	0.36 (0.03)	0.35 (0.03)	0.33 (0.03)	0.44	0.65	0.03
Fornix	0.36 (0.06)	0.33 (0.06)	0.30 (0.06)	0.30 (0.06)	0.01	0.45	0.29
**Association Fibers**							
Superior longitudinal fasciculus	0.41 (0.03)	0.39 (0.03)	0.41 (0.03)	0.38 (0.03)	0.46	0.001	0.37
Superior fronto-occipital fasciculus	0.39 (0.04)	0.34 (0.04)	0.37 (0.05)	0.34 (0.05)	0.64	0.001	0.48
Inferior fronto-occipital fasciculus	0.34 (0.02)	0.33 (0.02)	0.35 (0.02)	0.32 (0.02)	0.86	0.001	0.10
**Corpus Callosum**							
Genu	0.44 (0.03)	0.43 (0.03)	0.42 (0.03)	0.42 (0.04)	0.16	0.73	0.45
Body	0.50 (0.04)	0.47 (0.04)	0.49 (0.04)	0.46 (0.04)	0.19	0.007	0.67
Splenium	0.54 (0.03)	0.53 (0.03)	0.53 (0.03)	0.51 (0.04)	0.046	0.19	0.58

a Least squares means of FA values in PiB −/+ and WMH −/+ groups controlling for age, CDR, and presence of infarct.

a *p*-values from MANCOVA, df = 1,50.

Similar to the analysis in the entire study population, secondary analysis in the subset of subjects without infarct(s) revealed a marginal effect of PiB positivity on FA in the fornix (F_1,29_ = 3.29, p = 0.08) after controlling for age and CDR. WMH pathology was associated with lower FA in the corona radiata (F_1,29_ = 21.67, p<0.001), internal capsule (F_1,29_ = 14.56, p = 0.001), superior longitudinal fasciculus (F_1,29_ = 5.14, p = 0.03), and inferior fronto-occipital fasciculus (F_1,29_ = 5.38, p = 0.03), and marginally lower FA in the superior fronto-occipital fasciculus (F_1,29_ = 3.97, p = 0.06) and body of the corpus callosum (F_1,29_ = 3.89, p = 0.06). There was a positive interaction between PiB and WMH pathology on FA in the internal capsule (F_1,29_ = 4.24, p<0.05) and a marginally significant interaction in the parahippocampal white matter (F_1,29_ = 3.97, p = 0.06).

Table 4 summarizes results of the secondary analysis that accounted for apoE genotype. Because apoE genotyping was not available for all subjects, we first examined the effects of PiB status and WMH pathology in the subset of subjects with apoE genotype, controlling age, CDR, and presence of infarct(s). This revealed a main effect of PiB positivity on FA in the fornix (F_1,41_ = 4.84, p = 0.03) and in the splenium of the corpus callosum (F_1,41_ = 4.11, p<0.05); a main effect of WMH pathology on FA in the corona radiata (F_1,41_ = 24.33, p<0.001), internal capsule (F_1,41_ = 26.35, p<0.001), superior longitudinal fasciculus (F_1,41_ = 13.76, p = 0.001), superior fronto-occipital fasciculus (F_1,41_ = 8.00, p = 0.007), inferior fronto-occipital fasciculus (F_1,41_ = 7.95, p = 0.007), and body of the corpus callosum (F_1,41_ = 6.49, p = 0.02); and interactions between PiB and WMH pathology on FA in the internal capsule (F_1,41_ = 4.10, p<0.05) and parahippocampal white matter (F_1,41_ = 3.97, p = 0.05). In a second MANCOVA that controlled for apoE ε4 status in addition to age, CDR, and presence of infarcts, there was a main effect of PiB on fornix (F_1,40_ = 6.09, p = 0.02) and splenium (F_1,40_ = 5.23, p = 0.03) FA and a main effect of WMH pathology on FA in the corona radiata (F_1,40_ = 23.87, p<0.001), internal capsule (F_1,40_ = 25.91, p<0.001), superior longitudinal fasciculus (F_1,40_ = 13.51, p = 0.001), superior fronto-occipital fasciculus (F_1,40_ = 7.78, p = 0.008), inferior fronto-occipital fasciculus (F_1,40_ = 8.02, p = 0.007), and body of the corpus callosum (F_1,40_ = 6.65, p = 0.01). However, the interaction between PiB and WMH pathology in the internal capsule (F_1,40_ = 3.89, p = 0.06) and parahippocampal white matter (F_1,40_ = 3.91, p = 0.06) were no longer significant.

**Table 4:** Effects of PiB positivity and WMH pathology on FA in subset of subjects with apoE genotype^a.^

	p-value from MANCOVA^b^			Controlling for apoE^c^		
	PIB effect	WMH effect	PIB X WMH	PIB effect	WMH effect	PIB X WMH
**Projection Fibers**						
Corona radiata	0.45	<0.001	0.33	0.42	<0.001	0.34
Internal capsule	0.79	<0.001	0.049	0.72	<0.001	0.06
**Limbic Fibers**						
Cingulum	0.96	0.45	0.86	0.90	0.46	0.88
Parahippocampal WM	0.62	0.77	0.05	0.66	0.77	0.06
Fornix	0.03	0.90	0.30	0.02	0.93	0.26
**Association Fibers**						
Superior longitudinal fasciculus	0.57	0.001	0.66	0.73	0.001	0.62
Superior fronto-occipital fasciculus	0.78	0.007	0.14	0.92	0.008	0.15
Inferior fronto-occipital fasciculus	0.66	0.007	0.30	0.81	0.007	0.33
**Corpus Callosum**						
Genu	0.39	0.62	0.57	0.31	0.61	0.54
Body	0.28	0.01	0.64	0.20	0.01	0.59
Splenium	0.049	0.16	0.36	0.03	0.15	0.40

a PiB+WMH+: n = 13; PiB+WMH**−**: n = 14; PiB−WMH+: n = 14; PiB−WMH**−**: n = 16.

b controlling for age, CDR, and presence of infarct, df = 1,41.

c *p*-values from MANCOVA controlling for age, CDR, presence of infarct, and apoE ε4 status, df = 1,40.

## Discussion

In our examination of the effects of WMH pathology and Aβ deposition on white matter microstructural integrity, we found that (1) WMH pathology was significantly associated with reduced FA in projection and association fibers. (2) Aβ deposition was significantly associated with reduced FA in the fornix and splenium of the corpus callosum. (3) Although there were positive interactions between WMH pathology and Aβ deposition on FA in the internal capsule and parahippocampal white matter, accounting for apoE genotype rendered these interactions only marginally significant. Possible mechanisms and interpretations of the interaction are discussed below.

As hypothesized, WMH pathology was associated with reduced FA in projection and association fibers. This finding is consistent with earlier reports of an inverse relationship between FA and WMH volume [42], reduced FA in the presence of gliosis or infarcts [43], [44], extending beyond normal appearing white matter in subjects with cerebral amyloid angiopathy [45], cerebral autosomal dominant arteriopathy with subcortical infarcts and leukoencephalopathy (CADASIL) [46], and cognitive impairment with and without vascular dementia [47]. These results are also in agreement with prior reports that ischemic brain disease [48], [49] and vascular risk [15] alter FA in normal appearing white matter. The projection and association fiber tracts where we detected significant WMH pathology effects also correspond to the cerebral white matter regions with intermediate-high to low baseline anisotropy that Lee and colleagues [15] previously reported were most vulnerable to vascular risk.

As hypothesized, Aβ deposition was significantly associated with reduced FA in the fornix and selenium of the corpus callosum. Several studies have demonstrated that Aβ deposition can lead to DTI changes in mice [50], [51]. One previous study examined DTI changes associated with cerebral spinal fluid (CSF) Aβ levels in humans and found that Aβ42 levels were related to lower FA in medial frontal cortex and underlying white matter [52]. To the best of our knowledge, this is the first study to examine the relationship between cerebral Aβ deposition and FA. In the current study PiB positivity was associated with lower FA in the fornix, a predominant outflow tract of the hippocampus and a brain region affected early in the course of AD, consistent with previous DTI studies of MCI and AD [53]–[55]. PiB positivity was also associated with lower FA in the splenium of the corpus callosum, in accordance with previous ROI-based DTI studies of MCI and AD [56].

There is evidence suggesting that vascular brain injury exerts subtle deleterious effects on brain structure and function beyond frank infarction or hemorrhage [57]–[59]. For example, epidemiological studies have linked vascular risk factors (e.g., hypertension, hyperlipidemia, diabetes mellitus) to increased incidence of clinically-diagnosed AD [24], [25]. Therefore, we hypothesized that there would be an interaction between WMH pathology and Aβ deposition on FA. In the current study, we found an interaction between WMH pathology and PiB positivity in the internal capsule and parahippocampal white matter, where PiB-related reductions in FA was greater among participants who also had WMH pathology. However, these interactions were no longer significant after controlling for apoE ε4 genotype.

One possible mechanism by which WMH pathology and Aβ deposition might interact positively to degrade white matter microstructure is through the reduction of brain reserve. Reserve is the concept that there are characteristics of the brain that buffer the impact of pathology on brain performance [60], [61]. The characteristics may be structural (e.g., ‘extra’ neurons and/or synapses) or functional (e.g., a degree of excess capacity or compensatory mechanisms such that the brain could continue to perform well despite damage [62]). There is suggestive evidence that WMHs have a negative effect on structural brain reserve. For example, a population-based study of 1,077 non-demented elderly (60–90 years old) individuals found that greater periventricuar WMH severity was associated with a greater risk for developing dementia. Moreover, the association between WMH and dementia risk was independent of vascular risk factors, cerebral infarcts, and brain atrophy [63]. Hypertension, one of the strongest risk factor for WMHs, has also been linked with increased risk of developing AD [64] and treatment of hypertension has been found to be protective against the development of dementia [65]. Thus, in the context of the present study, it could be possible that WMH pathology was related to reduced structural brain reserve, which in turn rendered individuals with WMH pathology more vulnerable to the effects of cortical Aβ and AD pathology. Although it is not clear how precisely WMH pathology degrades brain reserve, some possible mechanisms include impaired neurogenesis and inflammation. Adult stem cells are located in the subventricular region [66], which is frequently affected by WMHs. Thus, the ischemic changes that lead to the development of WMHs could also cause damage to adult stem cells, impairing neurogenesis. Because there is microglial activation in brain tissue affected by WMHs [67] and higher C-reactive protein levels in patients with WMHs [68], WMH pathology may also negatively impact reserve through inflammatory cytokines. It is noteworthy that the interaction between WMH pathology and PiB positivity were no longer significant after controlling for apoE ε4 genotype. This suggests that another explanation for the interaction between WMH pathology and PiB may be apoE ε4. Presence of the apoE ε4 allele has been associated with increased amyloid deposition in the parenchyma [69] and amyloid positivity. Presence of the apoE ε4 allele may also be associated with amyloid deposition in the blood vessels, which could be associated withCAA and WMH pathology [70]. Future studies with a longitudinal design will be needed to examine if increased amyloid deposition in each of these regions is in fact associated with decreased white matter microstructural integrity in different regions of the brain.

The current findings should be considered in the context of several study limitations: First, the study sample was the small and heterogeneous. Second, apoE genotyping was only available in a subset of subjects. Therefore, our interpretation of the role of apoE in the interaction between PiB positivity and WMH pathology on FA are speculative at best and should be regarded with caution. Other study limitations include the low angular resolution of DTI (6 directions), the fact that we did not account for the location of vascular lesions in our analyses of FA values obtained from 9 white matter tracts that covered most of the supratentorial white matter, and the cross-sectional nature of the study design. Future studies with a longitudinal design will be able to better resolve the nature of the relationship between WMH pathology and PiB positivity on white matter microstructural integrity in different regions of the brain.
